# Inequalities in the spiritual health of young Canadians: a national, cross-sectional study

**DOI:** 10.1186/s12889-016-3834-y

**Published:** 2016-11-28

**Authors:** Valerie Michaelson, John Freeman, Nathan King, Hannah Ascough, Colleen Davison, Tracy Trothen, Sian Phillips, William Pickett

**Affiliations:** 1 Department of Public Health Sciences, Carruthers Hall, Queen’s University, Kingston, ON K7L 3 N6 Canada; 2 School of Religion, Queen’s University, Kingston, Canada; 3 Social Program Evaluation Group, Faculty of Education, Queen’s University, Kingston, Canada; 4 Dalhousie University, Halifax, Nova Scotia Canada; 5 Department of Emergency Medicine, Queen’s University, Kingston, Canada; 6 Department of Psychology, Queen’s University, Kingston, Canada

**Keywords:** Adolescent, Child, Determinants of health, Epidemiology, Health inequalities, Spiritual health

## Abstract

**Background:**

Spiritual health, along with physical, emotional, and social aspects, is one of four domains of health. Assessment in this field of research is challenging methodologically. No contemporary population-based studies have profiled the spiritual health of adolescent Canadians with a focus on health inequalities. In a 2014 nationally representative sample of Canadians aged 11–15 years we therefore: (1) psychometrically evaluated a series of items used to assess the perceived importance of spiritual health and its four potential sub-domains (connections with: self, others, nature and the natural environment, and the transcendent) to adolescents; (2) described potential inequalities in spiritual health within adolescent populations, overall and by spiritual health sub-domain, by key socio-demographic factors.

**Methods:**

Cross-sectional analysis of survey reports from the 2014 (Cycle 7) of the Canadian Health Behaviour in School-aged Children study (weighted *n* = 25,036). Principal components analysis followed by confirmatory factor analysis were used to explore the psychometric properties of the spiritual health items and the associated composite scale describing perceived importance of spiritual health. Associations among this composite scale, its individual sub-domains, and key socio-demographic factors were then explored.

**Results:**

The principal components analysis best supported a four-factor structure where the eight scale items loaded highly according to the original four domains. This was also supported in confirmatory factor analyses. We then combined the eight items into composite spiritual health score as supported by theory, principal components analysis findings, and acceptable tests of reliability. Further confirmatory factor analysis suggested the need for additional refinements to this scale. Based upon exploratory cross-sectional analyses, strong socio-demographic inequalities were observed in the spiritual health measures by age, gender, relative material wealth, immigration status, and province/territory.

**Conclusions:**

Study findings highlight potential inequalities in the spiritual health of young Canadians, as well as opportunities for methodological advances in the assessment of adolescent spiritual health in our population.

## Background

Spiritual health is recognized as the fourth domain of health, along with social, emotional, and physical dimensions [1–4]. In the pediatric field, there are benefits to including spiritual health as part of a holistic approach to the assessment of child health and wellbeing [5]. This view is congruent with the consideration of child spiritual health status as a basic human right, as included in the United Nations Convention on the Rights of the Child [6].

Despite recognition of the importance of spiritual health to children, there is incomplete consensus as to how it should be operationally defined [2, 7, 8]. What is established is that spiritual health represents a dimension of health that entails a condition of spiritual wellbeing. This involves some capacity for awareness of the sacred qualities of life experiences, and is typically characterized by “connections” in a range of subdomains, i.e., *connections to self* (internalized feelings and experiences), *to others* (externalized thinking and associated action), *to nature* (the natural environment), and *to the* “*transcendent*” (some sense of greater mystery beyond human experience) [9–11]. When connections within these four sub-domains are strong, positive aspects of spiritual health are experienced, which tend to be protective of overall health status [9–11].

There has been a recent surge in interest surrounding the application of spiritual health principles to clinical practice and also health promotion research in the field of paediatrics [12]. Clinical interventions are concerned with its application to hospital care, serious illness, and death [13, 14]. Within health promotion, positive health outcomes have been linked to interventions that are arguably spiritual in nature, including those involving exposure to nature [15], relaxation techniques and quieting exercises [16] and “mindfulness” strategies [12]. Our own Canadian research has identified strong relationships between the perceptions of the importance of spiritual health by children and many positive emotional health outcomes, including self-rated health status, low psychosomatic symptoms and high life satisfaction [17–19]. Positive spiritual health has the potential to be a significant health asset and a factor contributing to thriving among adolescents.

Despite its potential benefits, major gaps exist in the literature base surrounding the spiritual health of adolescents. Indeed, this topic has rarely been assessed in any sort of large-scale population-based study in our own country of Canada, and the field of adolescent spiritual health remains understudied more generally [20]. Assessment is challenging [8, 21] and epidemiological studies are rare and often related to individual spiritual health sub-domains. These include studies examining the importance of connections to nature [22] or connections to self [16].

We had a unique opportunity to address these gaps in knowledge. We conducted a national, population-based study in order to: (1) explore the psychometric properties of items that potentially contributed to a multidimensional, composite scale used to assess perceptions of the importance of spiritual health and its four potential sub-domains in adolescents; (2) describe potential inequalities in such perceptions, overall and by sub-domain, by key socio-demographic factors. Study findings provide foundational evidence in support of both clinical and health promotion efforts aimed at optimizing health in populations of young people, and provide direction for further methodological research in this emerging field.

## Methods

### Study populations and procedures

Health Behaviour in School-aged Children (HBSC) is a cross-national health promotion study affiliated with the World Health Organization [23]. It involves written health surveys conducted with students in classroom settings, with a focus on adolescents aged 11–15 years. In Canada, Cycle 7 of the HBSC was conducted in 2014 [24]. It involved participants in all Canadian provinces and territories. The national sample was stratified by province/territory, type of school board (public vs. separate), urban–rural geographic status, school population size, and language of instruction (French or English) with standardized population weights generated to account for over and under-sampling in some provinces and territories, and to ensure representativeness nationally by age group and gender. Participation was voluntary, and consent (explicit or implicit depending on local protocol) was sought from school administrators, parents, and participating students as per national human subject requirements. Participation of adolescents from private schools, home school situations, schools on First Nation or Inuit reserves, street youth not in school, and incarcerated youth was not sought. Youth not providing informed consent (explicit or implicit, as per local school board customs) were excluded. Ethics clearance was obtained from the Queen’s University General Research Ethics Board (Approval GMISC-062-13) and from the Health Canada/Public Health Agency of Canada.

Response rates were 100% at the provincial/territorial level and 77% at the individual student level. In total, 29,387 grades 6 to 10 young people participated. The sample was restricted to 25,321 young people with complete responses to the key variables of interest (age, sex, and the spiritual health module). Survey weights were applied to this sample, and the weighted sample used in subsequent descriptive analysis was 25,036 students (12,093 boys, 12,943 girls; Table 1). In Table 2, we restricted the analysis presented to a weighted sample of 11,375 grades 9–10 students (5,510 boys and 5,865 girls). There were small numbers of missing responses to the key items used in these sub-analyses (relative material wealth and immigration status), hence column totals do not sum to overall total. Table 3 provides analyses at the provincial and territorial level. Estimates were necessarily un-weighted, as it was only appropriate to apply survey weights to analyses based on the full national sample.

**Table 1:** Self-reported importance of adolescent spiritual health by age and gender, Canada, 2014, weighted *n* = 25,036

			Spiritual Health (Full Scale)	Spiritual Health Domain			
		Weighted		Self	Others	Nature	Transcendent
Gender	Age	n	Percentage of weighted n rated as important^a^ (row %)				
Boys	Total	*12093*					
	≤11	1120	63	84	80	70	44
	12	2057	57	83	79	66	36
	13	2363	51	81	76	62	34
	14	2604	43	80	71	54	31
	≥15	3949	39	75	72	49	28
	*p*_trend_^b^		<0.001	<0.001	<0.001	<0.001	<0.001
Girls	Total	*12943*					
	≤11	1185	70	86	85	76	48
	12	2268	65	85	82	71	42
	13	2504	55	79	82	64	37
	14	2929	49	80	79	56	32
	≥15	4057	46	82	82	53	28
	*p*_trend_^b^		<0.001	0.02	0.25	<0.001	<0.001

Note: (1) ^a^Score ≥8 out of 10 for individual domains, ≥32 out of 40 for full multidimensional scale; (2) ^b^Linear test for trend in percentages by age; (3) All analyses have been weighted

**Table 2:** Importance of specific domains of adolescent spiritual health by demographic subgroups, grades 9–10, Canada, 2014, weighted *n* = 11,375

		Weighted n	Spiritual Health Domain			
			Self	Others	Nature	Transcendent
Gender	Subgroup		Percentage of weighted n rated as important^a^ (row %)			
Boys	*By relative material wealth*:	*5356*				
	Not well off	406	67	68	43	26
	Average	1870	72	67.3	43	25
	Well off	3080	81	76	55	30
	*p*_trend_^b^		<0.001	<0.001	<0.001	0.04
	*By immigration status*:	*5463*				
	Born in Canada	4249	76	71	48	25
	Lived in Canada 6+ years	900	80	76	53	38
	Lived in Canada 1–5 years	314	80	75	61	45
	*p*_trend_^b^		0.07	0.09	<0.01	<0.001
Girls	*By relative material wealth*:	*5755*				
	Not well off	599	68	75	49	28
	Average	2215	78	77	51	25
	Well off	2941	8﻿7	86	55	32
	*p*_trend_^b^		<0.001	<0.001	0.10	0.02
	*By immigration status*:	*5812*				
	Born in Canada	4841	81	81	52	26
	Lived in Canada 6+ years	701	82	86	56	41
	Lived in Canada 1–5 years	270	87	83	64	56
	*p*_trend_^b^		0.06	0.07	0.01	<0.001

Note: (1) ^a^Score ≥8 out of 10 for each individual domain; (2) ^b^Linear test for trend by levels of subgroups; (3) All analyses have been weighted; and (4) Some columns do not total to the full sample size (*n* = 11,375) due to missing data on relative material wealth and immigration status

**Table 3:** Median percentages of adolescents reporting spiritual health as important within provinces and territories, Canada, 2014, un-weighted *n* = 25,321

	Percentage rated as important within the 13 provinces and territories^a^											
	Boys						Girls					
	Grades 6 to 8			Grades 9 to 10			Grades 6 to 8			Grades 9 to 10		
	(*n* = 6721)			(*n* = 5579)			(*n* = 7036)			(*n* = 5985)		
Indicator	Med	Min	Max	Med	Min	Max	Med	Min	Max	Med	Min	Max
*Overall Spiritual health score*	55	43	61	40	26	50	64	44	69	46	34	60
*By domain*:												
Connections to self	82	66	88	77	60	82	83	68	86	80	62	87
Connections to others	76	68	89	71	61	76	82	75	88	81	71	87
Connections to nature	64	60	73	52	43	60	70	59	76	54	46	69
Connections to transcendent	37	26	59	30	16	48	43	25	64	32	15	66

Note: (1) ^a^Score ≥8 out of 10 for each individual domain, ≥32 out of 40 for overall spiritual health score; (2) *Med* median, *Min* minimum, and *Max* maximum of the province/territory values for the percentage of students who rated as important, and; (3) Sample sizes for the provinces and territories ranged from 138 to 1352 for Grades 6 to 8 boys, 135 to 1454 for Grades 6 to 8 girls, 73 to 1198 for Grades 9 to 10 boys, and 65 to 1273 for Grades 9 to 10 girls

### Spiritual health

Measures used included eight simple questions that focused on the perceived importance of spiritual health in the lives of young people. This series of questions was adapted for brevity and literacy level from Fisher’s Spiritual Well-being scale [25], and focused on the perceived importance of spiritual health to young people as opposed to their lived experiences. Two items were asked for each of the four standard sub-domains. Students responded to these questions with one of five response categories ranging from 1- “*not at all important*” to 5- “*very important*.” The items asked students to identify at what level they thought it was important to: “*feel that your life has meaning or purpose*,” “*experience joy* (*pleasure*, *happiness*) *in life*” (connections to self); “*be kind to other people*,” “*be forgiving of others*” (connections to others); “*feel connected to nature*,” “*care for the natural environment*” (connections to nature); “*feel a connection to a higher spiritual power*,” “*meditate or pray*” (connections to the transcendent).

Factor analyses were conducted in SAS 9.4 (SAS Institute, Cary, NC) to explore the psychometric properties of the items, the four domains, then a potential spiritual health scale. We initially tested the 8-item module both quantitatively and qualitatively in Scotland and Canada in 2013. A Cronbach’s alpha value of >0.80 for the eight items was found in initial reliability testing. Focus group findings suggested that two items were not clearly understood by young people during these pilots, particularly in very young adolescents. Hence, the items were re-worded. We then tested this abbreviated and refined version of the module using the Canadian HBSC sample (*n* = 25,321), considering solutions with up to four factors. Principal components analyses involved *oblimin* rotation (which assumes correlation between items). Findings best supported a four-factor structure where the revised scale items loaded highly (each > 0.80) and according to the original four domains. This was further supported by a maximum likelihood goodness of fit test (*p* = 0.10), observed Cronbach’s alpha values of >0.80 for each of the four domains, and confirmatory factor analyses that supported a four-factor solution with fit statistics within acceptable ranges (RMSEA 0.06, SRMR 0.02, AGFI 0.97). This supported the conduct of analyses with the abbreviated 8-item scale but at the level of the four original domains. However, based on the original theoretical concept that would also support a composite measure of spiritual health, and a high degree of reliability (Cronbach’s alpha = 0.88), we also combined the 8 items into a single multi-dimensional scale, for exploratory purposes only.

### Other measures

Students reported their age in years, gender (*boy* or *girl*), school grade level (later stratified as *6 to 8*, *9 to* *10*), self-perceived relative measure of material wealth or advantage; *how well off to you think your family is*? (*1*-*Very well off*; *2*-*Quite well off*; *3*-*Average*; *4*-*Not very well off*; *5*-*Not at all well off*) [26], and immigration status (*born in Canada*, *immigrated 1*–*5 years ago*, *immigrated 6 or more years ago*). We also recorded the province or territory of residence for each participant.

### Statistical analysis

The main intent of our analysis was to profile potential inequalities in the perceived importance of spiritual health to young Canadians according to a number of socio-demographic factors. We also wished to explore methodologically our approach to the assessment of adolescent spiritual health at a population level. Our statistical approach was descriptive. Percentages of young Canadians rating aspects of spiritual health as important were described by age and gender. We then described relations between relative material wealth and spiritual health, and immigration status and spiritual health, with each analysis stratified by grade level and gender.

Cut-points for the exploratory spiritual health score were anchored on the response totals, with scores of 8 to 10 representing “*important*” for each of the four 2-item sub-domains, and 32 to 40 representing “*important*” for the exploratory 8-item scale. Tests for statistical significance in the linear trends of proportions were conducted by including the categorical variables as continuous predictors in log-binomial models, which accounted for clustering at the school level [27]. Variations between provinces and territories in the spiritual health scores were described using measures of central tendency. HBSC is 80% powered to identify absolute differences of 4% or higher with statistical significance (α = 0.05, 2 sided) within subgroups defined by age and gender, and <4% in the composite sample.

## Results

Girls on average reported spiritual health as being more important than did boys, both in the exploratory overall scale, and by sub-domain (Table 1). We also noted consistent declines in rated importance of spiritual health by age among both boys and girls. The most striking declines occurred for connections with nature and the natural environment, and connections with the transcendent. Based on the questions asked, both these sub-domains were reported as being important less frequently compared with connections with self and with others.

**Fig. 1:**
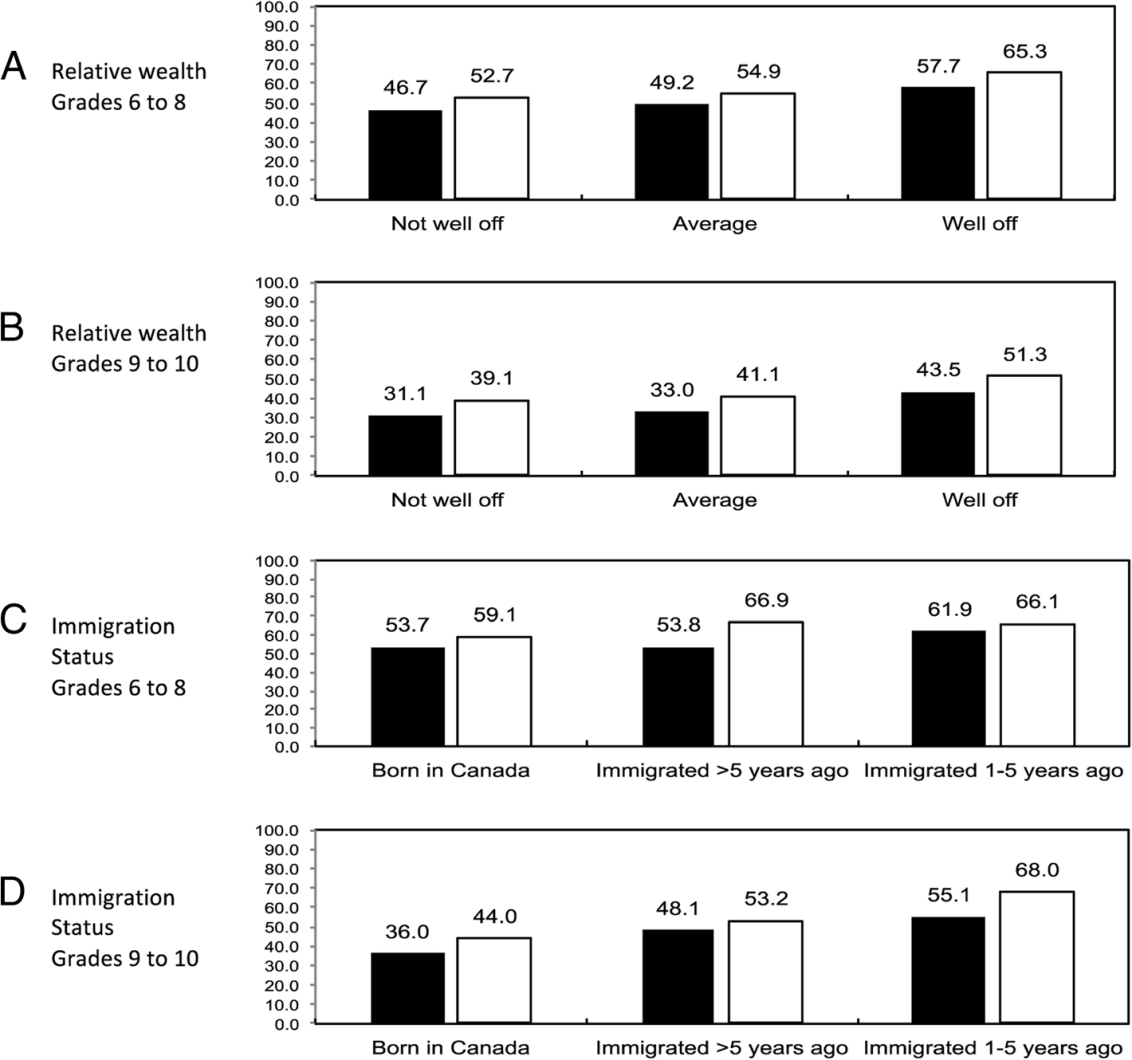
Young people reporting spiritual health as important by socio-demographic factors (level of relative material wealth (Panels **a** and **b**) and immigration status (Panels **c** and **d**)). *Black* Bars: Boys; *White* Bars: Girls. P_trend_ for each comparison <0.001

We noted strong and consistent patterns in the reported importance of spiritual health for relative material wealth and immigration status. As perceived relative wealth increased, the importance of spiritual health increased, both for the overall scale (Fig. 1) and for each sub-domain (Table 2). Lower percentages of young people rated spiritual health as being important among those who were born in Canada relative to recent immigrants.

Finally, we observed wide variations in the reported importance of spiritual health across 12 of the 13 provinces and territories (the spiritual health items were not asked in one province), with particularly large variations for the connections with “nature and the natural environment” and the “transcendent” domains (Table 3). As data were collected from provinces/territories with the explicit understanding that names of provinces and territories would not be identified, we omit these identifications here. We also examined variations in immigrant composition and material wealth (deprivation) across these jurisdictions as part of an assessment of potential confounding. Findings from a binomial regression analysis confirmed that some variation existed between the jurisdictions with respect to spiritual health; however, adjustment for relative material wealth and then immigration status had little or no impact on the effect estimates that summarized the inter-provincial and inter-territorial variations.

## Discussion

Adolescence is a key stage of life that requires ongoing focus as children learn, grow and develop. Society pays great attention to almost all aspects of the health of young people during adolescence, with physical, mental, and social health the subject of a wide range of well-intended preventive interventions [28, 29]. However, although recognized by the WHO and many Indigenous cultures as a fourth domain of health [1], and by UNICEF as a fundamental human right [6], the spiritual health of young people in Canada has not been a significant focus for research and intervention development and even more rarely has it been quantified empirically. This lack of attention represents an important gap in the biomedical and social science literatures.

The most important findings of this national study were as follows. *First*, we adapted a series of measures in an attempt to describe and quantify the perceived importance of spiritual health to adolescents. This series of items was brief and at an appropriate level of literacy for children as young as 11 years of age. The content of this module was informed by theory [2, 3, 8] and the analyses presented with these items are unique to the Canadian adolescent health literature. The overall scale, while exploratory, shows promise as a composite measure of some key components of adolescents’ rating of the importance spiritual health, as opposed to their lived reality of spiritual health experiences. Further refinement of this scale is also indicated. *Second*, we applied these items, both as a composite scale and then by each of the four spiritual sub-domains, to the study of inequalities in spiritual health in young Canadians. In doing so, we demonstrated inequalities by age, gender, relative material wealth, immigration status, and geography. This profile is unique to a literature that is dominated by theoretical discussions and qualitative enquiry [30] and provides new evidence that is helpful to our Canadian context.

Our methodological exploration in this field of research is important. While recognized as a concept that is best measured in composite [2, 3, 8], assessment of spiritual health as a multi-dimensional construct is uncommon [25] with less psychometric research and very few credible quantitative studies in early adolescence [20] particularly in our own country of Canada. Our findings show the possibilities of adapting a brief, factor-analytically derived scale to early adolescence that is theoretically sound and considers the four sub-domains of spiritual health multi-dimensionally. While further refinement to this scale is desirable, findings from our principal components analyses were promising, meeting all conventional criteria for scale development [31]. We also found that this measure had very reasonable confirmatory psychometrics, although it performed best as a 4-factor solution rather than a composite scale, and these findings resonate with those of a substantial body of qualitative work with children that provided the theoretical basis for its development [17]. The number of items included (eight) was also the maximum permitted by the national research collaboration involved in the Canadian HBSC survey. Moving forward, scale refinements might involve reverting to the larger number of items present in Fisher’s original scale [25], and consideration too of lived experiences of spiritual health (i.e., whether or not young people experience this themselves) to compliment our existing ratings of its perceived importance.

We were able to demonstrate strong and consistent inequalities in self-reported importance of spiritual health, both overall and by sub-domain, in association with all versions of the scale derived in our analysis. Such inequalities have not been quantified previously nor examined in terms of how they influence wellbeing, development, and other aspects of adolescent health. The highly gendered patterns that were observed, for example, may reflect the ways that boys and girls are differentially socialized in Canadian society. Because boys and girls may relate to the four established sub-domains of spiritual health differently, gender-specific approaches to the promotion of healthy connections, relationships, and other aspects of spiritual health are warranted.

The reported declines in perceptions of importance of spiritual health related to age may reflect normative changes in cognition, reason, abstract thought, and independent thinking that come naturally with adolescent growth and development [32]. More challenging, such declines may relate to the deeper emotional needs that emerge during adolescence, and thus also relate to the mental health of young people. Our developmental findings point to a persistent demand to promote and foster healthy relationships in adolescent lives. Relationships lie at the heart of what it is to be a human being [33]. Healthy connections, whether they are within adolescents themselves, with others in their lives, or with the world that surrounds them, relate strongly to their health and their ability to flourish [34]. We believe that the quality of these connections lies at the heart of the concept of adolescent spiritual health, and that this is some of what is being reflected through this measure.

Our analysis also identified inequalities in reported perceptions of the importance of spiritual health in association with indicators of the social environments that surround young Canadians. We demonstrated these inequalities for a measure of relative material wealth (which showed that young people who perceived themselves to be less well off attached less importance to spiritual health), immigration status (those born in Canada provided lower ratings for its importance vs. recent immigrants), and geographic status (wide variations existed across the provinces and territories).

While any self-reported adolescent health survey will be limited by its reliance on the subjective perceptions of adolescents with respect to socio-demographic factors (e.g., our measure of relative material wealth) as well as our indicators of spiritual health, our findings still have merit. They were, however, different than evidence presented in some past studies. Lower socio-economic status has been shown to correlate directly with known risks for health, and self-perceptions have also been shown to be more consistent determinants of health than measures of lived experience [35–37]. Lower levels of perceived socio-economic status also have been found to correlate strongly with higher levels of religiosity in both children and adults [38], consistent with the “deprivation theory,” which posits that poor individuals are more likely to be religious than those who are materially wealthy [39]. This past evidence, however, only focuses on the correlation between religious attitudes and expression, and lower socioeconomic status, and does not account for the broader protective qualities of spirituality that lie outside of religious involvement. When one views the broader adolescent literature on spirituality, different findings emerge. For example, “poor teenagers are less likely than non-poor teenagers to report meaningful experiences of spiritual worship” [40], while youth who report low socio-economic status also report low levels of existential well-being [41]. With respect to the transcendental domain, ours, and past findings, indicate a need for scholars to distinguish between the concepts of spirituality and religiosity, as their social patterns may in fact be quite different for adolescents.

Our findings do reinforce the idea that the origins of spiritual health are in part cultural, and perhaps reflect the values and tenets of the social environment. The geographic findings may indicate that jurisdictions with educational policies and programs that bring spiritual practices (e.g., mindfulness inspired activities, relationship-building programs and outdoor education initiatives) into the school setting are potentially facilitating the development of positive spiritual health. Past findings [17–19, 42] have also demonstrated that measures such as ours describing spiritual health are strongly associated with the health status of young people, whether that is measured in composite or via specific indicators of mental and emotional health. More in-depth investigation of the mechanisms by which spiritual health is promoted and optimized in specific social contexts is warranted, as higher levels of spiritual health coincide with healthier relationships and associated positive health outcomes that help young people to thrive.

Canada, like many other countries, is experiencing an epidemic of mental health problems in its young people. There is not one comprehensive explanation for the failure of our children and adolescents to thrive. The cultural contexts in which such health inequalities have arisen have been the subject of debate and scrutiny [43]. Extensive work by Louv and others suggests that health inequalities may be attributed to what we are presenting as the third sub-domain of spiritual health, a disconnection from the natural world due to a lack of intentional exposures to such environments [15]. Other cultural explanations include the intense pace and expectations of modern life for children [44] and consumerism [45]. In turn, there has been a recent surge in interest around adolescent spiritual health and its application to these modern day challenges, both clinically and in terms of primary prevention [12–14]. Optimization of spiritual health has been related to positive health outcomes including happiness among children [30], as well as resilience [46]. Spiritual health may indeed be an under-appreciated positive health asset to the health of young people.

Canadian political scientists have argued that Canada is best defined in terms of its regional variations, with some authors emphasizing provincial/territorial distinctions by legal boundaries [47, 48] and others downplaying these boundaries [49]. These variations have come about through migration patterns from different cultures and the balance between urban/rural populations, among other factors. The provincial/territorial cultures are expressed in voting patterns and political views. The analyses in this paper indicate the possibility that the variations extend to views on aspects of spiritual health. Speculatively, this may be congruent with the greater individualism in some regions and the greater collectivism in other regions.

Strengths and limitations of this study warrant comment. Our analysis is novel and addresses some fundamental gaps in the adolescent health and spiritual health research literatures. The analysis was large and national in scope. Our efforts to adapt and test a quantitative, composite measure of the perceived importance of spiritual health advances attempts to foster research in this field, while our demonstration of potential health inequalities points to avenues for health promotion and clinical intervention. Limitations include our recognized need for further refinement of the adolescent spiritual health scale, measurement error inherent to self-report surveys, and limits on causal inference attributable to the cross-sectional nature of our study opportunity. The potential for reverse causality for our focal relationships of interest is clearly possible.

## Conclusions

This national study explored potential social inequalities spiritual health and its four domains among young people in Canada. In addition to adapting a series of indicators to be used in the assessment of the perceived importance of spiritual health by adolescents, strong socio-demographic inequalities were observed in the spiritual health measures by age, gender, relative material wealth, immigration status, and province/territory. The current analysis represents the first of many steps in an emerging research program. Our findings point to the need for improved assessment surrounding the concept of adolescent spiritual health. They point to the need for deeper research, both qualitative and quantitative, to understand the mechanisms by which inequalities in adolescent spiritual health emerge, as well as the importance of such inequalities, and possibly inequities, to the health of young people in Canada and internationally.

## Abbreviations

AGFI: Adjusted goodness of fit index
HBSC: Health behaviour in school-aged children
RMSEA: Root mean square error of approximation
SRMR: Standardized root mean square residual
